# Strengthening Delivery of Health Services Using Digital Devices

**DOI:** 10.9745/GHSP-D-18-00229

**Published:** 2018-10-10

**Authors:** Maeghan Orton, Smisha Agarwal, Pierre Muhoza, Lavanya Vasudevan, Alexander Vu

**Affiliations:** a Department of Reproductive Health and Research, World Health Organization, Geneva, Switzerland.; b Johns Hopkins Global Digital Health Initiative, Baltimore, MD, USA.; c Johns Hopkins Bloomberg School of Public Health, Baltimore, MD, USA.; d Department of Community and Family Medicine, Duke University School of Medicine, Durham, NC, USA.; e Center for Health Policy and Inequalities Research, Duke Global Health Institute, Durham, NC, USA.; f Johns Hopkins School of Medicine, Baltimore, MD, USA.; g School of Medicine, American University of Beirut, Beirut, Lebanon.

## Abstract

**Background:**

Delivery of high-quality efficient health services is a cornerstone of the global agenda to achieve universal health coverage. According to the World Health Organization, health service delivery is considered good when equitable access to a comprehensive range of high-quality health services is ensured within an integrated and person-centered continuum of care. However, good health service delivery can be challenging in low-resource settings. In this review, we summarize and discuss key advances in health service delivery, particularly in the context of using digital health strategies for mitigating human resource constraints.

**Methods:**

The review updates the foundational systematic review conducted by Agarwal et al. in 2015. We used PubMed, EMBASE, and CINAHL to find relevant English-language peer-reviewed articles published 2018. Our search strategy for MEDLINE was based on MeSH (medical subject headings) terms and text words of key articles that we identified a priori. Our search identified 92 articles. After screening, we selected 24 articles for abstract review, of which only 6 met the eligibility criteria and were ultimately included in this review.

**Results:**

Despite encouraging advances in the evidence base on digital strategies for health service delivery, the current body of evidence is still quite limited in 3 main areas: the effectiveness of interventions on health outcomes, improvement in health system efficiencies for service delivery, and the human capacity required to implement and support digital health strategies at scale. Two particular areas, digital health-enhanced referral coordination and mobile clinical decision support systems, demonstrate considerable potential to improve the quality and comprehensiveness of care received by patients, but they require a greater level of standardization and an expanded scope of health worker engagement across the health system in order to scale them up effectively.

**Conclusions:**

Additional research is urgently needed to inform the effectiveness of interventions on health outcomes, improvement in health system efficiencies, and cost-effectiveness of service delivery. In particular, more documentation and research on ways to standardize and engage health workers in digital referral and clinical decision support systems can provide the foundation needed to scale these promising approaches in low- and middle-income settings.

## Abstract (precis)

Delivery of high-quality efficient health services is a cornerstone of the global agenda to achieve universal health coverage. Digital health interventions for service delivery, such as digital health-enhanced referral coordination and mobile clinical decision support systems, demonstrate considerable potential to improve the quality and comprehensiveness of care received by patients but require greater standardization and engagement of health workers at different levels of the health system for effective scale up.

## INTRODUCTION

Delivery of high-quality efficient health services is a cornerstone of the global agenda to achieve universal health coverage. According to the World Health Organization (WHO) framework of health system building blocks, health service delivery is considered to function well when equitable access to a comprehensive range of high-quality health services is ensured within an integrated and person-centered continuum of care.^1^ However, good health service delivery can be challenging in settings where human and health system resources are scarce. For instance, health workers in low-resource settings may be faced with inadequate access to training and reference materials, poor-quality communication systems for feedback from experts or supervisors in the diagnosis and management of complex cases, and difficulty maintaining patients within the continuum of care through follow-up visits or referrals, thereby impacting the quality of health services they can deliver.^2^

Similarly, accessing health services, according to individuals' needs and preferences, at the various levels of the health system may be challenging due to logistical and financial barriers. In recent years, the considerable mobile-cellular infrastructure has been leveraged to mitigate some of these challenges in health service delivery, for instance, by facilitating task shifting of health service delivery from facility-based providers to frontline health workers.^3^ In these task-shifting applications, mobile devices have been used to provide training content on-demand, enable communication between different cadres of health workers, implement clinical decision support systems, and provide work-planning and scheduling tools. There is growing evidence that such ‘digital health’ strategies can help improve access to and quality of health service delivery, which, in turn, can improve health outcomes for otherwise underserved populations.^4^ Although rural–urban differences in access to and uptake of mobile technology remain, significant progress toward increasing universal access has been made. For instance, it is estimated that global mobile–broadband subscription growth rates grew more than 20% annually in the last 5 years and are expected to reach 4.3 billion by the end of 2017.^5^

In this review, we summarize and discuss key advances in health service delivery, particularly in the context of using digital health strategies for mitigating human resource constraints. We focus the discussion on clinical decision tools and digital referral systems and how recent innovations within these areas have contributed to improvements in health service delivery. We also analyze and discuss gaps in the current evidence base on the effectiveness of the digital health service delivery interventions on health care use, efficiency, and outcomes. Finally, we provide recommendations for and highlight challenges in scaling up digital health service delivery strategies within health systems.

## METHODS

The purpose of this literature review is not to serve as a comprehensive systematic review of all relevant published articles but rather to identify important new evidence on digital strategies for health service delivery that may advance the current body of knowledge and practice. The scope of the review was based on the framework on integrated patient-centered health services.^6^ This framework includes 5 potential areas of strategic focus.^6^ Digital health interventions, which support a model of care (strategy 3, mobile clinical decision support systems) and coordination of health services (strategy 4, digital referral systems), are discussed in this review. Other reviews in this supplement describe digital health interventions for demand generation (strategy 1) and governance (strategy 2). For this review, we included peer-reviewed studies from high-, middle-, and low-income countries, which described implementation and evaluation of digital strategies for improving health service delivery (Figure).

The scope of the literature review was based on 5 potential areas for strategic focus from the WHO framework of integrated patient-centered services.

Our review is based on the foundational systematic review done by Agarwal et al. in 2015.^7^ Our search strategy incorporated the key search terms from the Agarwal et al. review, which included variations and combinations of terms for mHealth (mobile, phone, cell phones, information and communication technology, cellular phone, mobile device, SMS, text message, interactive voice response) and health workers (frontline worker, health worker, community health worker, traditional birth attendants, lay worker, village health worker, midwife, health auxiliary, peer health worker, medical auxiliary, health provider, lay advisor, lay counselor, lady health worker, and lay educator).^7^ To this, we added variations and combinations of key search terms for service delivery (health service, service availability, service readiness, health facilities, service quality, service coverage, service coordination). We then updated the review via a literature search using the databases on PubMed, EMBASE, and CINAHL for relevant publications published between 2015 and 2018. We restricted our searches to studies published in English and developed a search strategy for MEDLINE based on MeSH (medical subject headings) terms and text words of key articles that we identified a priori.

The updated search resulted in 92 peer-reviewed articles. One of the authors screened the articles and identified 24 abstracts for final review. Two of the authors worked independently and in duplicate to review titles, abstracts, and full-text versions of the identified articles. The inter-rater agreement was 92%. The discrepancy was with 2 articles. After a face-to-face discussion, the reviewers agreed that the 2 studies should be excluded because the studies reported on feasibility and pilot study protocols that did not add to the body of evidence about health care delivery using digital health. Only 6 articles met the eligibility criteria for the review.

## RESULTS AND DISCUSSION

### Landscape of State of Evidence on Digital Strategies for Health Service Delivery

There are a number of published studies with rigorous study designs and reporting (i.e., randomized controlled trials, prospective cohort studies, and detailed study protocols), diversity of intervention strategies tested, and selection of appropriate evaluation indicators. Interventions described in these studies cover the spectrum of health service delivery, and include education (training in use of mobile phones for health delivery),^8^^–^^15^ diagnosis and management of diseases (mobile clinical decision support systems and referral coordination),^16^^–^^29^ communication between health care providers,^30^^–^^35^ and communication between provider and health care consumers (appointment reminders and test-result notification).^36^^–^^40^ While this is not a comprehensive review, notable landmark articles on the use of digital strategies for health service delivery are described and referenced below:

- Several studies reported effective ways to use mobile phones to collect and report data from frontline health workers to health delivery teams, thus bypassing the need for in-person communication for data transfer. Client data were then used by the health delivery team to engage direct patient care by, for example, sending clients health messages or reminders to their mobile phones with the aim of improving health education and behavior change communication.^41^^,^^42^
- Lori et al.^12^ conducted a study on the training of trainers to train community midwives on the use of short messaging service (SMS) messages for real-time remote data collection in rural Liberia. The study reported a significant increase in overall knowledge and skill acquisition among the 99 traditional midwives who used mobile technology for SMS-based data collection.^12^
- Zurovac et al.^29^ conducted a cluster-randomized trial on the effects of SMS message reminders on health workers in Kenya. The results showed that health workers who received SMS message reminders had significantly improved (23.7%) adherence to malaria treatment guidelines compared to the control group who did not receive SMS message support.^29^
- Kim et al.^43^ used SMS and web-based systems to achieve glycemic control with significantly improved glycated hemoglobin (HbA1c) in the intervention group compared to the control group in a randomized controlled clinical trial in Korea.^43^
- Similarly, Goodarzi et al.^44^ conducted a randomized controlled clinical trial in Iran using SMS messages to educate patients with diabetes about exercise, diet, medication compliance, and self-monitoring of blood glucose. Results showed statistically significant improvement in HbA1c levels, diet, physical activity, knowledge, and self-efficacy among the intervention group compared to the control.^44^
- Mitchell et al.^24^ showed digital decision-making tools significantly improved adherence to the Integrated Management of Childhood Illness (IMCI) protocol among health providers who used electronic decision-support tools in Tanzania. A few other studies yielded similar promising results, suggesting that mHealth can improve communication and supervision of health workers and evaluate health workers' performance.^26^^,^^42^^,^^45^

The results of the updated literature search revealed additional noteworthy high-quality studies with greater use of objective measures and rigorous research methodology:

- Lim et al.^46^ conducted a randomized controlled clinical trial in Korea to achieve glycemic control using a clinical decision support system and physical activity monitoring device and dietary feedback among patients with type 2 diabetes. After 6 months, HbA1c levels were substantially improved, with a significantly improved decrease in caloric intake and increase in exercise among the intervention compared to the control group.^46^
- Agboola et al.^47^ conducted a randomized controlled clinical trial and used SMS messages to coach and monitor patients with type 2 diabetes with HbA1c levels of >7 to achieve physical activity goals. There was no significant difference in change of HbA1c levels or monthly step counts in the 6-month follow-up between the intervention compared to the control group.^47^ Arora et al.^48^ and Capozza et al.^49^ also used SMS in randomized controlled clinical trials to educate, motivate, and achieve glycemic control, however they showed no statistical improvement in HbA1c. All of these studies were conducted in high-income countries.
- Daher et al.^50^ conducted a systematic review and meta-analysis of 99 studies published from 1996 to 2017, and found that SMS interventions improved antiretroviral therapy adherence with pooled odds ratio (OR) of 2.15 (95% confidence interval [CI], 1.18 to 3.91) and clinic attendance rates with pooled OR of 1.76 (95% CI, 1.28 to 2.42). However, the authors did acknowledge that misclassification bias and recall bias were high (58% bias among randomized controlled trials and 64% among quasi-randomized trials) and raised concern regarding the quality of studies included in the meta-analysis.^50^

Despite these encouraging advances in the evidence base on digital strategies for health service delivery, much of the literature is still focused on descriptive data or intervention potential. A substantial number of current studies used self-reported outcomes related to health behaviors, management, or service delivery or use. Only a few studies used objective measures of health or health service delivery.^7^^,^^43^^,^^44^^,^^46^^–^^49^^,^^51^^–^^53^

Despite encouraging advances in the evidence base on digital strategies for health service delivery, much of the literature remains focused on the use of descriptive data or intervention potential rather than objective measures.

### Mobile Clinical Decision Support Systems

As described previously, health providers from low-resource settings face multiple barriers to the delivery of high-quality efficient health services. These barriers may include: health care providers' limited access to timely and relevant health information; a shortfall of appropriately trained health care workers, especially in rural and remote areas; and the consequential transfer of responsibility for primary health care service delivery to lay health care workers who have little to no health service-related training.^54^ Even in settings where health care workers may have adequate training, it may be difficult for them to learn of new evidence and apply it consistently and correctly across a range of disease groups. Mobile clinical decision support systems (CDSS) have potential to mitigate these barriers. CDSS is an “electronic system” designed to aid directly in clinical decision making, in which the characteristics of individual patients are used to generate patient-specific assessments or recommendations that are then presented to clinicians for consideration.^55^ The concept behind CDSS is not novel. Clinical decision support tools have been used in hospital-based settings with varying levels of sophistication in high-income countries for several decades.^56^ However, employing CDSS on mobile devices can provide opportunities for such tools to become available in areas with limited infrastructure and outside of hospital- or clinic-based settings. As task shifting from a higher cadre of trained providers to lay health workers is increasingly supported, mobile CDSS (mCDSS) can provide novel opportunities to continually train and support these lay health workers.

Providing CDSS on mobile phones may serve a range of functions, including guiding health care providers through process algorithms using *if…then* rules based on evidence-based protocols, providing a checklist based on extant clinical protocols, or providing step-by-step guidance to screen clients by risk status using predetermined models. An mCDSS application may be stand-alone—to be used at a single point in time to deliver services—or may be integrated with a longitudinal health record, where any information that is entered into the system at a single point in time can be retrieved and used for making decisions during a follow-up visit. Systems that combine mCDSS with health records can facilitate long-term care and support the appropriate referral of clients at different levels of the health system. For example, an intervention developed in partnership with the Tanzanian Ministry of Health and Social Welfare provided community health workers with a mobile job aid to counsel, screen, and provide health-facility referrals to women at the community level for pregnancy, sexually transmitted infections, and family planning services. The data collected during these routine community-level service-provision visits were stored in electronic forms and sent to a central server that could be accessed by district-level health staff.^57^ This type of intervention improves not only the quality and comprehensiveness of services provided by lay health workers at the community level but also facilitates appropriate linkages to care and management at the facility level.

mCDSS can guide health care providers through process algorithms, provide a checklist based on extant clinical protocols, or provide step-by-step guidance to screen clients by risk status.

The evidence in support of the use of mCDSS is slowly emerging. A before–after cluster trial in Tanzania provided frontline health workers with a mobile electronic decision-support tool to assess sick children according to IMCI protocols. The study reported a significant improvement in the providers' ability to adhere to 10 critical IMCI items.^24^ Most studies conducted in low-income settings focus on the feasibility of such interventions and lack a high level of rigor to assess the impact of mCDSS on the quality of health services and health outcomes.^7^^,^^58^ However, some conclusions may be drawn from interventions conducted in high-income countries. A review conducted by Free et al.^51^ identified 7 trials that provided mobile support in clinical diagnosis and management to providers across 25 outcomes. Of the 25 outcomes, 19 showed benefits, of which 11 were statistically significant. The remaining 6 outcomes showed negative effects related to increased time for clinical processes or errors in data. However, none of the trials were assessed to have a low risk of bias.^51^

While mCDSS tools are promising, the challenge of transitioning from paper-based health records and decision-support tools to digital systems must not be underestimated. Despite efforts to make the mCDSS user-interface accessible and user friendly, the learning curve to adopt digital systems is often steep and requires ongoing training and support. To function well, such systems need to be iteratively developed, take into account user feedback, and align closely with existing clinical protocols.

The learning curve to adopt digital systems can often be steep and requires ongoing training and support.

The broader challenge of long-term adoption and scale up is how to ensure digital records are considered official records by ministries of health. As digital systems are tested, managers of health systems are often reluctant to dispose of existing paper systems. The result is that health care workers are then required to enter the same information in both paper and digital systems, adding to the responsibilities of the already overworked frontline health workers. Appropriate efforts must be undertaken to prove that digital records are as or more accurate than paper records. While mCDSS may be promising, the adoption of digital systems relies on understanding whether these systems can work in their specific context or environment, and if the systems can be effectively rolled out at scale.

### Digital Referral Systems

Digital referral systems enable client health needs to be managed in a comprehensive manner using resources beyond those available at the patient's initial access to care. When referral activities are delivered effectively, patients are able to receive the full scope of care that is available from their health system, regardless of their geographic location.^59^^,^^60^ In practice, referral management and coordination include the following activities:

1. Identifying the signs during a clinical encounter that a referral is needed
2. Preparing the client for this referral
3. Arranging logistics to transport the client to the location of referral
4. Ensuring receipt of health services according to client need at the referral facility
5. Managing receipt of the client at the returning facility where relevant

Digital referral systems enable client health needs to be managed in a comprehensive manner using resources beyond those available at the patient's initial access to care.

It is important to note that referral management and coordination is not an isolated process. It is embedded within the context of proper diagnosis, patient support, and post-treatment follow up.^59^^,^^60^ When these related processes are inadequate, they can impact the effectiveness of referral systems. In health systems that are still reliant on paper-based data collection systems, there is a limitation to the degree in which patient referrals can truly be coordinated: paper referral forms can get lost, delays in paper-based information arriving at the right level of care may occur, and low levels of literacy can create challenges in comprehension.^61^ Furthermore, the failure of patients to complete the full care plan in line with their initial diagnosis can often be attributed to a breakdown of referral processes.^62^^,^^63^ The reasons behind these breakdowns can be complex and multifactorial: referrals to clinic may not account for distance clients need to travel, clients may not be able to afford the means to travel to the clinic, clients may not be able to afford taking time off from work or have child care arrangements to be able to follow up with the clinic, and the client or caregiver may not understand the referral instructions. Enhancing referral coordination activities with digital health systems can help overcome substantial barriers to strengthening referral services.

Within the body of research included in this review is a wide array of digital health referral coordination systems that focus on health domains ranging from maternal and child care to noncommunicable diseases and dental care. The primary users of the digital referral systems in these studies included community health workers, clinical officers, nurses, and medical doctors. Several articles describe the improved effectiveness of digital referral systems over the standard of care. For instance, in Zambia, researchers reported a marked improvement in referrals for patients as a result of using coordinated digital health referral coordination systems.^62^ In addition, the digital referral system removed barriers to arranging referrals faced by health care providers by improving the providers' ability to communicate with others, preparing patients for care, and changing plans for referral activities quickly, if needed. Similarly, in Zanzibar, the authors noted that an increased proportion of women completed the recommended 4 antenatal care visits, leading researchers to believe that digital health interventions could contribute toward the overall improvement of maternal health.^64^ These findings present a strong case to assess the feasibility of scaling referral system.

Even with the implementation of digital referral systems, several challenges related to data completeness remain that limit our ability to assess the effectiveness of these systems. Standard reporting formats typically provide a limited assessment of referrals as a health performance indicator. For instance, referrals are often recorded based on their status (e.g., as complete or incomplete), without providing the details of the nature of the referral, completion of the counter referral, or outcome for the patient. The lack of detailed information prevents an accurate assessment of the quality of health service delivery to the patient. In other cases, referral data may be binary, only counting referrals made, and, occasionally, referrals completed. These types of data sets fail to provide the information required to understand the impact of digital referral systems on improving service delivery, health outcomes, and, importantly, health systems strengthening activities aimed at achieving universal health coverage.

Binary referral data fail to provide the information required to understand the impact of digital referral systems on improving service delivery, health outcomes, and health systems strengthening activities.

The limited choice of available software and lack of standardization, in terms of data collection and integration, also poses a significant challenge to scaling digital referral systems.^62^ The studies in this review deployed referral systems using different and noncompatible digital health software, and none of the systems collected data in the same format.^62^^,^^64^^–^^68^ In some cases, these divergent approaches were implemented in the same country, resulting in unnecessary duplication and limiting opportunities for integration and scale up. Additionally, the literature review revealed gaps related to how referral systems engage patients along the full continuum of care—from the point of initial contact to treatment and management. Two studies included in this review focused on community health workers as the primary referral points for patients and tracked whether patients arrived for treatment at the next level of the health system.^62^^,^^68^ However, they provided limited to no information about whether the health workers at the next level of care received the patient successfully or whether the treatment was provided to the patient as intended. For example, in Uganda, researchers conducted a detailed review of the number of children who were referred for treatment for malnutrition. The study's metric for success was the overall number of referrals completed during the duration of the study.^62^^,^^68^ These metrics, however, failed to capture a clear picture of the patient's engagement with the health system. Hence, future implementers and evaluators of digital referral systems must consider how to generate data that are beneficial for quality improvement and not limit themselves to proximal indicators in the pathway to care.

Despite the increased proliferation of mobile phones and affordability of mobile broadband technology in low- and middle-income countries, only about 30% of the global rural population currently has mobile phone access, compared to approximately 90% of the urban population,^69^ and this level of unique mobile-cellular subscriptions is insufficient to support universal access. These trends are particularly important to note when considering the delivery of health services through digital health programs. Ultimately, for digital health strategies to be instrumental in the achievement of universal health coverage goals, a better understanding and stronger emphasis on how they can be used to deliver large-scale, timely, and comprehensive health services to both rural and urban populations will be required.^70^ The Table summarizes key published articles that discuss digital referral systems and mobile clinical decision support systems.

For digital health strategies to be instrumental in the achievement of universal health coverage goals, we need to better understand how they can be used to deliver large-scale, timely, and comprehensive health services to both rural and urban populations.

## Conclusions

The current body of evidence on digital strategies for health service delivery is still quite limited in 3 main areas: the effectiveness of interventions on health outcomes, the improvement of health system efficiencies for service delivery, and the level and type of human capacity required to implement and support digital health strategies at scale.^7^^,^^51^ Additional research is urgently needed to inform these gaps and to show the cost-effectiveness of digital health interventions to provide and support service delivery. Digital health interventions for service delivery, such as digital health-enhanced referral coordination and mCDSS, demonstrate major potential to improve the quality and comprehensiveness of care received by patients. However, these digital health interventions require a greater level of standardization to prepare for scale and an expanded scope of health worker engagement to include more levels of health service delivery. These specific enhancements, if researched and documented, can provide the foundation needed to scale effective digital referral coordination and decision support systems within low- and middle-income settings.

**FIGURE:**
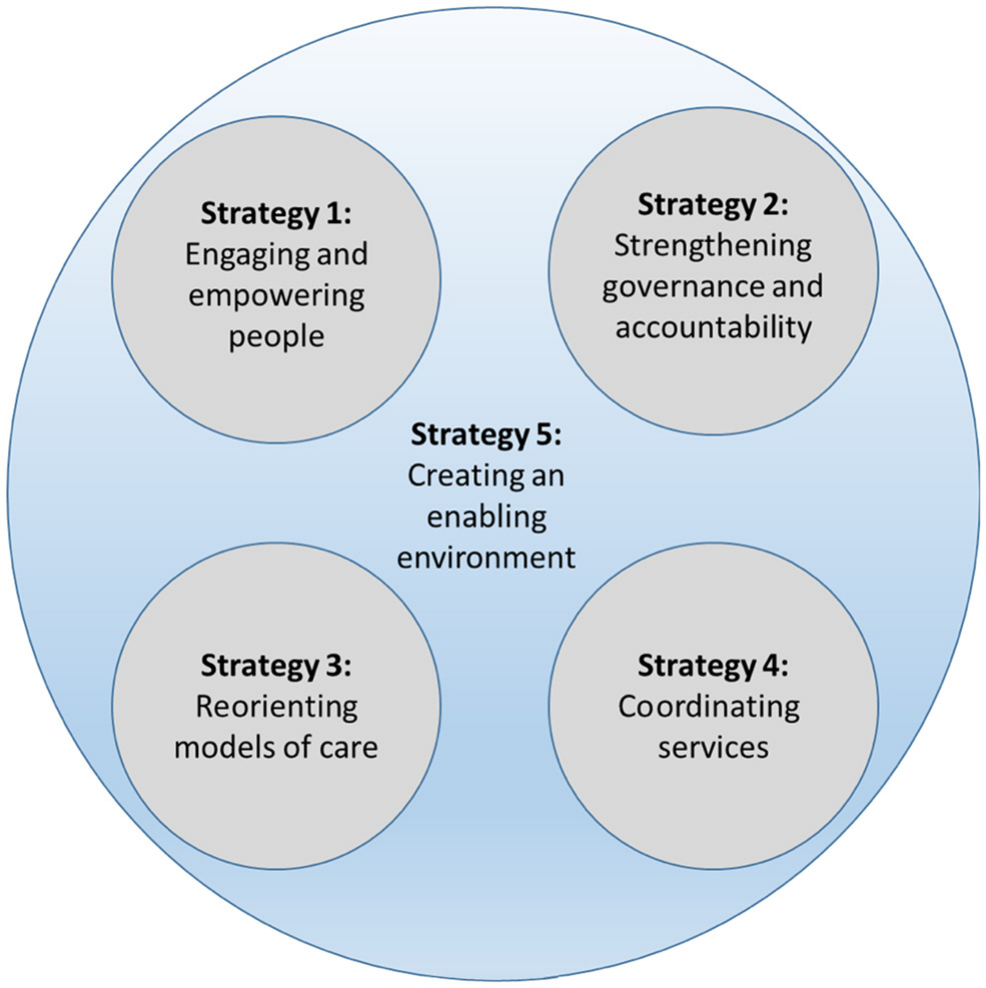
Strategic Focus Areas of the Integrated Patient-Centered Health Services Framework

**TABLE.** Major Findings and Limitations of Published Studies on Digital Referral Systems and Mobile Clinical Decision Support Systems

Author(s), Year of Publication	Major Findings	Limitations
Agarwal et al., 2015^7^	The authors demonstrated that mobile job aids can help CHWs deliver integrated counseling on family planning and HIV/STI screening and collect relevant programmatic data on service delivery.	Study is not able to show whether collected data was of good quality and usable by decision makers.
Agboola et al., 2016^47^	This randomized controlled trial examined the effect of personalized text messages on physical activity, as measured by a pedometer, and clinical outcomes in patients with diabetes. Patients who received the SMS messages had significantly higher monthly step counts in the third (RR=4.89; 95% CI, 1.20 to 19.92) and fourth (RR=6.88; 95% CI, 1.21 to 39.00) months of the study compared to the control group. However, over the 6-month follow-up period, monthly step counts did not differ statistically by group. HbA1c levels decreased by 0.07% (95% CI, 0.47 to 0.34) in the intervention group compared to the control group.	Operational challenges related to pedometer software installation and Internet access to upload activity data contributed to a high attrition rate in the study. Investigators also noted differential rates of activity tracker adherence across comparison groups. Group differences in baseline HbA1c that could potentially bias comparisons of follow-up changes were also observed. Finally, the study did not evaluate the effectiveness of the different types/themes of messages.
Capozza et al., 2015^49^	The authors used a randomized controlled trial design to assess the impact on glycemic control of a 2-way SMS-based intervention that provided daily behavioral coaching, education, and testing reminders to patients with diabetes. The secondary aim of the study was to examine patient interaction and satisfaction with the program. The study was conducted in the context of a 6-month clinic-based quality improvement initiative. A comparison of the intervention group and the controls (who continued their usual care without receiving SMS messages) showed similar decreases in average HbA1c levels after 90 and 180 days of follow up, probably reflecting the success of the broader quality improvement initiative. Almost a third (29%) of program users in the intervention group demonstrated frequent engagement, and survey results indicated very high satisfaction with the program.	The primary outcome, change in HbA1c, is difficult to affect in the short time frame (6 months), and sample size was small (58 and 35 in intervention and control groups, respectively). Study also reported wide variation in the timing of baseline HbA1c measures relative to study onset. Authors also reported difficulties recalling patients to the clinic for regular HbA1c testing.
Daviaud et al., 2017^68^	The authors conducted an economic analysis of the implementation of ICCM, which includes the integrated diagnosis, treatment, and referral services for malaria, suspected pneumonia, and diarrhea among children by CHWs. The analysis was conducted across 6 African countries and assessed country-level scale-up implications. Their analysis indicated that between 10 and 603 treatments were given per CHW per year. Weighted economic costs per treatment ranged from US$2 to US$13. CHWs spent from 1 to 9 hours a week on ICCM.	The paper focused on annual costs to providers (health system and donors) to inform planning and budgeting but did not assess program effectiveness due to the recentness of program implementation. CHW time on the program was based on the same assumptions of length of visit and meetings for all countries rather than on observation. Authors note that even though implementation costs are calculated on an annual basis, recent guidelines recommend using a wider window of time.
den Hollander and Mars, 2017^67^	The authors conducted a retrospective review of a referral database of cell phone-generated images to demonstrate that telemedicine can be a reliable method of triaging patients before admission into a burn unit. In 66% of studied cases, telemedicine consultation avoided inappropriate admission or delayed admission in late referrals until the patient was ready for definitive treatment.	Study highlighted complex issues related to patient data security and confidentiality.
Dobson et al., 2017^52^	The systematic review examined 7 randomized controlled trials that investigated the use of SMS-based self-management interventions for patients with diabetes. No clear relationship between positive outcomes and intervention dose, content, and functionality was observed.	The small number of articles reviewed was due, in part, to inclusion criteria restricting studies to randomized controlled trial designs. Because only published full-text papers in English were included, the study results were potentially influenced by publication and language bias.
Kabakyenga et al.,2016^62^	Findings from this observational study suggest that using mobile phones to support the implementation of ICCM by CHWs could improve supportive care for acutely ill children.	The study's design and limited sample size of only 96 trained CHWs did not allow a full assessment of demonstrable improvement in health outcomes attributable to mobile-phone support.
Lim et al., 2016^46^	This clinical trial randomized patients with diabetes into either a group offering routine diabetes care with self-monitored blood glucose or a group employing an Internet-based monitoring device that provided real-time individualized feedback (u-healthcare) system combined with exercise monitoring and dietary feedback. The investigators examined the effect of the u-healthcare combination intervention on glycemic control. After 6 months of follow up, the HbA1c level was significantly decreased in the u-healthcare group (8.0% ± 0.7%) compared with the SMBG group (8.1% ± 0.8 %; *P*<.01).	The study was limited to individuals with access to mobile phones and Internet. Additionally, the 6-month follow-up period may not be long enough to evaluate the long-term effect of this system.

Abbreviations: CHWs, community health workers; CI, confidence interval; HbA1c, hemoglobin A1c; ICCM, integrated community case management; RR, risk ratio; SMBG, self-monitored blood glucose; SMS, short message service; STI, sexually transmitted infection; u, ubiquitous.

## Notes

**Peer Reviewed**

**Funding:** This work was made possible by a grant from the Aetna Foundation, with support from the World Health Organization and The Johns Hopkins University Global mHealth Initiative. In addition, Vasudevan's effort on this publication was supported in part by the National Center for Advancing Translational Sciences of the National Institutes of Health under Award Number 1KL2TR002554.
